# Nonparametric estimation of conditional survival function with time-varying covariates using DeepONet

**DOI:** 10.1007/s10985-026-09700-6

**Published:** 2026-03-24

**Authors:** Bingqing Hu, Bin Nan

**Affiliations:** Department of Statistics, University of California, Irvine, CA 92697 USA

**Keywords:** ADNI study, Brier score, Convolutional neural networks, Cox model, Deep operator networks, Feedforward neural networks

## Abstract

Traditional survival models often rely on restrictive assumptions such as proportional hazards or instantaneous effects of time-varying covariates on the hazard function, which limit their applicability in real-world settings. We consider the nonparametric estimation of the conditional survival function, which leverages the flexibility of neural networks to capture the complex, potentially long-term non-instantaneous effects of time-varying covariates. In this work, we use Deep Operator Networks (DeepONet), a deep learning architecture designed for operator learning, to model the arbitrary effects of both time-varying and time-invariant covariates. Specifically, our method relaxes commonly used assumptions in hazard regressions by modeling the conditional hazard function as an unknown nonlinear operator of entire histories of time-varying covariates. The estimation is based on a loss function constructed from the nonparametric full likelihood for censored survival data. Simulation studies demonstrate that our method performs well, whereas the Cox model yields biased results when the assumption of instantaneous time-varying covariate effects is violated. We further illustrate its utility with the ADNI data, for which it yields a lower integrated Brier score than the Cox model.

## Introduction

Survival prediction is of increasing importance, and estimating the conditional survival function from censored data nonparametrically is of great interest. Recent advances in machine learning and deep learning have overcome certain limitations of traditional semiparametric survival analysis methods (Cox 1972; Wei 1992), such as Gradient Boosting Machines (GBM) (Hothorn et al. 2005; Ridgeway 1999) and Random Survival Forests (RSF) (Ishwaran et al. 2008), which are ensemble methods based on survival trees (Leblanc and Crowley 1993); and DeepSurv (Katzman et al. 2018) and DeepHit (Giunchiglia et al. 2018), which are based on neural networks. Although some of them (Hothorn et al. 2005; Ridgeway 1999; Katzman et al. 2018) take more flexible non-linear forms, they still keep certain model structures in the framework of either the Cox model or the accelerated failure time (AFT) model. Also, they estimate a risk score to summarize the features rather than estimating the conditional survival function directly. DeepHit divides the continuous time into a series of evenly partitioned time intervals and outputs the probability of an event occurring in each time interval. RSF is a flexible approach that uses the logarithmic rank test to split the data and directly output the survival probabilities at the observed time points. However, these methods are primarily developed for time-invariant covariates, although extensions are possible for handling time-varying covariates. In particular, Cygu et al. (2023) found that the computational cost is extremely high for RSF when dealing with time-varying covariates.

Following the same data expansion technique used to fit a Cox model, Hu and Nan (2023) proposed a nonparametric approach using neural networks to directly estimate the conditional survival function given time-varying covariates. It takes time-varying covariates, baseline covariates, and a corresponding time interval of a partition formed by all observed time points as input to a feedforward neural network (FNN) and the logarithm of the conditional hazard function as output. The method uses a loss function determined by the nonparametric full likelihood, thus does not assume any specific model structure.

To the best of our knowledge, all known methods for time-varying covariates, such as those discussed above, assume that the effects of covariates on the conditional hazard function are instantaneous. That is, the instantaneous hazard given entire covariate paths depends only on the values of covariates observed at the current time point. Such an assumption can be unrealistic. Considering flexible non-instantaneous effects of time-varying covariates on the hazard function can be of great interest and importance in addressing such issues, for example, when the hazard is related to an arbitrary cumulative exposure or certain delayed covariate effects. A nonparametric approach for non-instantaneous covariate effects provides the most flexible way of estimating how covariates influence survival, which captures complex temporal dynamics and eliminates model biases in survival prediction.

In this work, we further generalize the method of Hu and Nan (2023) to account for arbitrary effects of entire covariate histories of time-varying covariates. The time-varying covariates are functional inputs and the conditional survival function (or equivalently the conditional hazard function) becomes an operator. We propose applying the recently developed deep operator network, DeepONet (Lu et al. 2021a), to estimate the unknown operator. To our knowledge, the method is the first to model arbitrary effects of time-varying covariates in estimating the conditional survival function nonparametrically with the fewest assumptions.

## Methodology

Consider survival times of *n* subjects, which can be right-censored. We are interested in estimating the survival time distribution given a set of covariates, and some or all of them are time-varying covariates. For subject *i*, we denote the time-varying covariate vector as $X_i(t)$. We further denote the underlying failure time as $T_i$, and the underlying censoring time as $C_i$. We assume that the failure time possesses a Lebesgue density and that the censoring time is independent of failure time given covariates. Let the observed time be $Y_i = \min \{T_i, C_i\}$ and the failure indicator be $\varDelta _i = I(T_i\le C_i)$. The observations $\{Y_i,\varDelta _i,\widetilde{X}_i(\cdot ): \ i=1,\dots ,n\}$ are independent and identically distributed, where $\widetilde{X}_i(t)$ denotes the covariate history up to time *t*, that is, $\widetilde{X}_i(t)=\{X_i(s), 0\le s \le t\}$. Let $f[t | \widetilde{X}_i(\infty )]$ and $f_C[t|\widetilde{X}_i(\infty )]$ be the conditional density functions of $T_i$ and $C_i$, respectively; and $S[t|\widetilde{X}_i(\infty )]$ and $S_C[t|\widetilde{X}_i(\infty )]$ be the conditional survival functions of $T_i$ and $C_i$, respectively.

We further assume that future covariate values do not influence the current risk. In other words, the risk of the event of interest occurring at time *t* only depends on the current and past values of covariates, not on future values, so the covariates are external. Otherwise, the time-varying covariate is called the internal covariate (Kalbfleisch and Prentice 2002). Note that the conditional survival probability is not well-defined if there is an internal covariate. Then the conditional hazard function of $T_i$ can be written as:

1 $$\begin{aligned} \lambda \left[ t \left| \widetilde{X}_i(\infty )\right. \right] =\lambda \left[ t \left| \widetilde{X}_i(t)\right. \right] . \end{aligned}$$

To meet the positivity constraint for the hazard function, we transform the hazard function with the logarithm to ease numerical implementations. Denote

$$\begin{aligned} h[t, \widetilde{X}_i(t)] = \log \lambda [t|\widetilde{X}_i(t)]. \end{aligned}$$

Then the conditional cumulative hazard function given covariate history has the following form:

$$ \varLambda \left[ t \left| \widetilde{X}_i(\infty )\right. \right] = \varLambda \left[ t \left| \widetilde{X}_i(t)\right. \right] =\int _{0}^t \lambda \left[ s \left| \widetilde{X}_i(s)\right. \right] ds=\int _{0}^t e^{h[s,\widetilde{X}_i(s)]}ds, $$

and the conditional survival function is given by

2 $$\begin{aligned} S\left[ t \left| \widetilde{X}_i(\infty ) \right. \right] = \exp \left\{ -\varLambda \left[ t \left| \widetilde{X}_i(t)\right] \right. \right\} = \exp \left\{ -\int _{0}^t e^{h[s,\widetilde{X}_i(s)]}ds \right\} . \end{aligned}$$

Equation (2) is always a valid survival function for any function $h[s,\widetilde{X}_i(s)]$. Once the function *h* is estimated, we can easily determine the conditional survival function estimator.

### Data structure and DeepONet

Clearly $h[s,\widetilde{X}_i(s)]$ is an operator since $\widetilde{X}_i(s)$ can be viewed as a functional input. We propose to estimate $h[s,\widetilde{X}_i(s)]$ nonparametrically using the DeepONet (Lu et al. 2021a). Following Lu et al. (2021a), we partition the time axis evenly into *m* intervals at $t_0\equiv 0< t_1< t_2< \cdots < t_m\equiv \tau$ and consider covariate values on these grid points, where *τ * is a finite upper bound of the considered support of the survival time, often taken to be the largest observed failure time in a dataset in practice. Note that $h[s,\widetilde{X}_i(s)] = h[s,\widetilde{X}_i(\tau )]$ for external covariates $X_i(\cdot )$, we input only the historical values of the covariates up to the time point *s* and mask future values with 0. This turns out to be a valid approach to apply the DeepONet, which can be seen from the result of the universal approximation theorem for operators given in Eq. (3). Then following Hu and Nan (2023) we expand the observed covariates $x_i(\cdot )$ of each subject *i* into *m* rows of inputs, as shown in Table  1.

**Table 1:** The data structure for time-varying covariates

*i*	Time point	Covariates
1	$t_1$	$x_1(t_0),\, 0,\, \dots ,\, 0$
1	$t_2$	$x_1(t_0),\,x_1(t_1),\,0,\, \dots ,\, 0$
$\vdots\,\,\,\,$	$\vdots\,\,\,\,$	$\vdots\,\,\,\,$
1	$t_m$	$x_1(t_0),\, x_1(t_1),\, \dots ,\, x_1(t_{m-1})$
2	$t_1$	$x_2(t_0),\, 0,\, \dots ,\, 0$
2	$t_2$	$x_2(t_0),\, x_2(t_1),\, 0,\, \dots ,\, 0$
$\vdots\,\,\,\,$	$\vdots\,\,\,\,$	$\vdots\,\,\,\,$
2	$t_m$	$x_2(t_0),\, x_2(t_1),\, \dots ,\, x_2(t_{m-1})$
$\vdots\,\,\,\,$	$\vdots\,\,\,\,$	$\vdots\,\,\,\,$

**Fig. 1:**
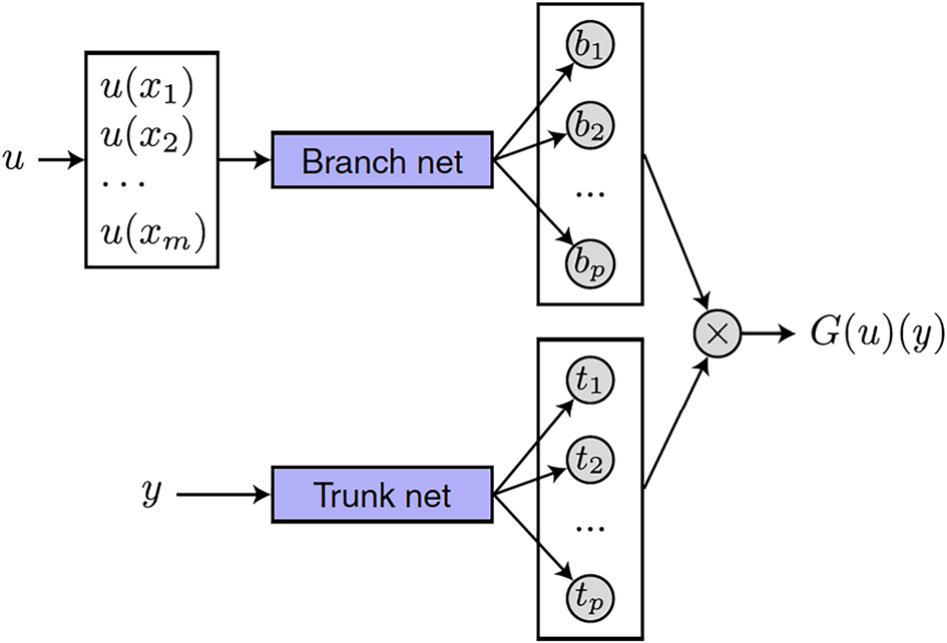
The unstacked DeepONet Structure. *u*, *y*, *G*(*u*)(*y*) in this figure correspond to *x*, *s*, *h*(*x*)(*s*), respectively

To implement the DeepONet, we adopt a classical neural network architecture, either a fully connected feedforward network (FNN) or a convolutional neural network (CNN), and use the concatenation of the current time *s* and the covariate history up to time *s*, $\widetilde{x}_i(s)$, as the network input. Note that this is different to the existing work that only models instantaneous covariate effect, in which the network input is the concatenation of *s* and the covariate value at *s*, i.e., $[s, x_i(s)]$ (Biganzoli et al. 1998; Giunchiglia et al. 2018; Hu and Nan 2023; Islam et al. 2024). Our approach naturally leads to the formulation of the conditional survival distribution as an operator. Recently, using neural networks to approximate operators has drawn increasing attention in the literature (Lu et al. 2021a; Kovachki et al. 2021). Lu et al. (2021a) showed that the deep operator network can learn various explicit operators, such as integrals and fractional Laplacians, as well as implicit operators that represent deterministic and stochastic differential equations. Our problem can be seen as an operator learning problem. Using the operator notation, we denote $h[s,\widetilde{x}_i(s)]$ as $h[\widetilde{x}_i(s)](s)$ and use both of them exchangeably in this article. Specifically, we use an unstacked DeepONet structure to estimate $h[\widetilde{x}_i(s)](s)$. A DeepONet consists of two sub-networks – the branch net for encoding the input function at *m* sensors $[x(t_0), x(t_1),..., x(t_{m-1})]^T$ and the trunk net for encoding the location *s* for the output function (Lu et al. 2021a). See Fig. 1 for an illustration where the input is denoted as *(u(· ), y)* and the output is denoted as *G*(*u*)(*y*).

The architecture of branch and trunk neural networks is inspired by the universal approximation theorem for operators (Chen and Chen 1995). The theorem states that two fully connected neural networks with a single hidden layer, combined by a vector dot product of the outputs, are able to approximate any continuous nonlinear operator with arbitrary accuracy. Specifically, suppose that *σ * is a continuous non-polynomial function, *X* is a Banach space, $K_1 \subset X$ and $K_2 \subset {R}^d$ are compact sets in *X* and ${R}^d$, respectively, *V* is a compact set in $C(K_1)$, and *G* is a nonlinear continuous operator which maps *V* into $C(K_2)$. Here *C*(*K*) is the Banach space of all continuous functions defined on *K* equipped with the uniform norm. For any *ε > 0*, there are positive integers *n*, *p*, and *m*, constants $c_i^k$, $\xi _{ij}^k$, $\theta _i^k$, $w_k \in {R}$, $x_j \in K_1$, *i = 1, … , n*, *k = 1, … , p*, and *j = 1, … , m*, such that

3 $$\begin{aligned} \left| G(u)(y) - \sum _{k=1}^p \sum _{i=1}^n c_i^k \sigma \left( \sum _{j=1}^m \xi _{ij}^k u(x_j) + \theta _i^k \right) \sigma (w_k \cdot y + \zeta _k) \right| < \epsilon \end{aligned}$$

holds for all *u ∈ V* and $y \in K_2$.

A generalized version of the approximation theorem (Theorem 2 in Lu et al. (2021a)) states that for any nonlinear continuous operator *G* and any *ε >0*, there exist positive integers *m*, *p*, continuous vector functions $g: {R}^m \rightarrow {R}^p$, $f: {R}^d \rightarrow {R}^p$, and $x_1, x_2, \ldots , x_m$, such that,

4 $$\begin{aligned} |G(u)(y)-\langle g[u(x_1),u(x_2),...,u(x_m)],f(y)\rangle | < \epsilon \end{aligned}$$

holds for all *u* and *y*, where $\langle \cdot , \cdot \rangle$ denotes the dot product in ${R}^p$. The functions *g* and *f* can be chosen as diverse classes of neural networks, for example, FNN or CNN.

### Full likelihood and discretized loss

We propose using the full likelihood to build the loss function. Given observations $\{y_i,\delta _i,x_i(\cdot )\}$, the full likelihood function is

$$\begin{aligned} L_n= & \prod _{i=1}^n \left\{ f[y_i | \widetilde{x}_i(y_i)]S_C[y_i | \widetilde{x}_i(\infty)]\right\} ^{\delta _i}\left\{ f_C[y_i | \widetilde{x}_i(\infty)]S[y_i | \widetilde{x}_i(y_i)]\right\} ^{1-\delta _i} \nonumber \\\propto & \prod _{i=1}^n \lambda [y_i |\widetilde{x}_i(y_i)]^{\delta _i}S[y_i |\widetilde{x}_i(y_i)] \nonumber \\= & \prod _{i=1}^n \exp \{h[\widetilde{x}_i(y_i)](y_i)\delta _i\}\exp \left\{ -\int _0^{y_i} e^{h[\widetilde{x}_i(t)](t)}dt \right\} . \end{aligned}$$

The log-likelihood is given by

5 $$\begin{aligned} \ell _n = \sum _{i=1}^n \left\{ h[\widetilde{x}_i(y_i)](y_i)\delta _i-\int _0^{y_i} e^{h[\widetilde{x}_i(t)](t)}dt\right\} . \end{aligned}$$

By calculating the above integrals numerically using the summation on a partition of *[0, τ ]* with *m* subintervals, we obtain the loss function that is the discretized negative log-likelihood, scaled by the sample size:

6 $$\begin{aligned} loss(h)&= \frac{1}{n} \sum _{i=1}^n \sum _{j=1}^m I(t_{j-1} \le y_i) \Big \{ e^{h[\widetilde{x}_{i}(t_{j-1})](t_{j-1})}(t_j - t_{j-1}) \\ & \quad - \, h[\widetilde{x}_i(t_{j-1})](t_{j-1}) \delta _{ij}\Big \}, \nonumber \end{aligned}$$

where $\delta _{ij} = I(t_j > y_i)\delta _i$. Note that $\delta _{ij}$ is always 0 until the event happens.

### Details in network architectural and hyperparameter tuning

We implement the DeepONet using the python package (Lu et al. 2021b). The package is primarily for solving ordinary and partial differential equations. It supports five tensor libraries as backends. To adapt the method to our problem, we modify the code in DeepXDE using its TensorFlow 2.x backend, including using our customized loss function and modifying the branch net structure for the CNN method. We train the neural networks on a system equipped with an AMD Ryzen 9 8945HS processor with Radeon 780 M Graphics at 4.00 GHz and 32GB of RAM.

For the trunk net, we use the simple one layer FNN structure because there is only one scalar input of time. For the branch net, we use either FNN or CNN to summarize the time-varying covariates, where the FNN has two fully connected hidden layers (a.k.a. dense layer), and the CNN has two blocks of 1D convolutional layers (Kiranyaz et al. 2021) and max pooling layers followed by one fully connected layer. When using the FNN structure for the branch net, we concatenate the time-invariant covariates to the time-varying covariates directly; when using the CNN structure, however, it is not reasonable to concatenate two types of covariates so we first input the time-varying covariates into CNN and then concatenate the time-invariant covariates with the CNN output before going through the final fully connected layer. The masked future values of 0 for the time-varying covariates (see Table 1) will not contribute to the output of the FNN branch net because the multiplication with those future time input-weights will be 0, regardless of the weight values (see Eq. (3)). To ensure that future covariate values do not contribute to the output of the CNN branch net, we use casual padding (Van Den Oord et al. 2016) that automatically neglects the future time points. Our simulations given in the next section show that the CNN branch net performs better, where we consider one time-varying covariate and two time-invariant covariates. This is not surprising because CNN preserves the temporal neighborhood information of the time-varying covariates.

We use an independent validation dataset to decide when to stop during training and use another independent test dataset to tune the hyperparameters. Specifically, in each training, we stop when the validation loss no longer decreases, and we choose the hyperparameter combination with the smallest test loss. In our simulations, we choose the hyperparameters from the following combinations:

- number of nodes in each dense layer: [32, 64, 128, 256]
- number of filters in each Conv1D layer: [16, 32, 64]
- pool size: [4, 8]
- learning rate: [0.01, 0.001, 0.0001]
- batch size: [100, 500, 1000]

## Simulations

We generate data from a model that has cumulative covariate effects on the conditional hazard function. Thus the Cox model under the usual assumption $\lambda [t|\widetilde{X}_i(t)] = \lambda [t|X_i(t)]$ is misspecified in this setup.

First, we generate a time-varying covariate on a fine grid of [0, 100]. For $t \in \{0, \varDelta s, 2\varDelta s,....,100\}$ with *\varDelta s = 0.1*, $i \in \{1,2,...n\}$, we generate random variables $\alpha _{i1},\dots ,\alpha _{i5}$ independently from the $\textrm{Uniform} \, (0,1)$ distribution and construct a time-varying covariate as the following:

$$\begin{aligned} X_{i}(t) &= \alpha _{i1}+\alpha _{i2}\sin (2\pi t/\tau )+\alpha _{i3}\cos (2\pi t/\tau )+\alpha _{i4}\sin (4\pi t/\tau ) \\ & \quad + \alpha _{i5}\cos (4\pi t/\tau ). \end{aligned}$$

The sample path $x_i(t)$ of the above covariate is a left-continuous step function with right limit on the grid. Then we generate two time-invariant covariates $Z_{i} \sim \textrm{Bernoulli} \, (0.5)$ and $W_{i} \sim \textrm{Normal} \, (0,1)$. For a set of covariate values, we determine the following conditional hazard function of the survival time:

$$\begin{aligned} \lambda (t | \widetilde{x}_i(t), z_i, w_i) &= 0.05\exp \Bigg (w_i+z_i+0.01\sum _{s\le t} x_i(s)\varDelta s \\ & \quad + 0.01\sum _{s\le t} x_i^2(s) z_i\varDelta s \Bigg ), \end{aligned}$$

then evaluate the conditional cumulative hazard function and the conditional survival function on the grid:

7 $$\begin{aligned} \varLambda (t |\widetilde{x}_i(t), z_i, w_i) &= \varDelta s \sum _{s\le t}\lambda (s | \widetilde{x}_i(s),z_i,w_i), \end{aligned}$$

8 $$\begin{aligned} S(t |\widetilde{x}_i(t), z_i, w_i) = \exp \left\{ -\varLambda (t | \widetilde{x}_i(t),z_i,w_i)\right\} . \end{aligned}$$

To generate the failure time $T_i$, we first generate $U_i$ from the $\textrm{Uniform}\, (0,1)$ distribution, then set $T_i=\sup \left\{ t:S(t |\widetilde{x}_i(t), z_i, w_i) \ge U_i\right\}$. We generate the censoring time $C_i$ by taking the minimum of a randomly generated value from the $\textrm{Exponential} \, (50)$ distribution and 99 which yields around 20% censoring rate. Then we have $Y_i = T_i \wedge C_i$ and $\varDelta _i=I(T_i\le C_i)$.

Note that such generated time variable takes discrete values on the fine grid, which can be viewed as a numerical approximation of the continuous time variable. But due to its discrete nature, the exact survival function should be expressed as a product integral determined by the above hazard function (7). We find, however, the above survival function given in (8) approximates the exact survival function adequately in our simulation studies. Using the product integral in (8) provides a way to analyze discrete survival times.

We independently generate training sets and validation sets each with a sample size of *n=2000*, then use the time-varying covariate values taken on *m* equally spaced grid points on [0, 100] to train the DeepONet. We treat *m* as another hyperparameter that is selected from the set $\{50, 100, 200, 300, 400, 500\}$. We first generate 10 independent data sets to tune *m* while fixing other hyperparameters to a commonly used baseline configuration (number of nodes in each dense layer: 64, number of filters in each Conv1D layer: 32, pool size: 4, learning rate: 0.001, batch size: 500). For each of these 10 data sets, we identify the value of *m* that minimizes the validation loss. We then fix *m* at its median value of 250 obtained from those 10 optimal values and use *m=250* in subsequent simulations. In practice, we suggest applying cross-validation for selecting the optimal *m*. We then tune the other hyperparameters using an independent dataset, which gives the following values that are used in simulation runs: the number of nodes in each dense layer (except for the last layer in the branch and trunk net) is 128, the number of filters in each Conv1D layer is 16, the pool size is 8 with the same stride, the learning rate is 0.001, and the batch size is 1000. We have observed that the neural network hyperparameters are not very sensitive in our simulations. “Relu" function is used as the activation function between the hidden layers, and linear function is used for the final output so that the output value is not constrained. “Adam" is used as the optimizer. In the last layer before doing the dot product, we use *p=10* nodes as suggested in Lu et al. (2021a). Finally, we use the fitted model to estimate the conditional survival curves given four different sets of newly generated covariates. We repeat the process *N=200* times, plot the sample average and 90% empirical pointwise confidence bands of the conditional survival curves on these four sets of covariates.

**Fig. 2:**
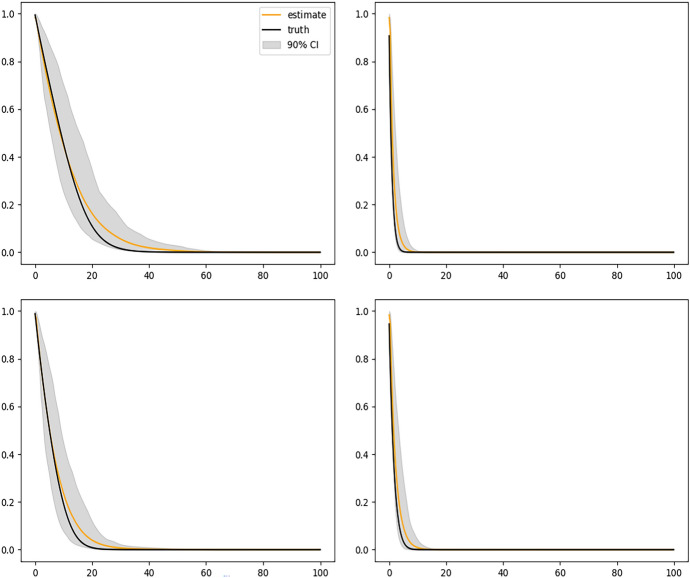
Conditional survival curves for 4 different sets of covariates estimated by the DeepONet with FNN branch net and sample size *n = 2000*. In each plot, black solid curve is the true conditional survival curve; the yellow solid curve is the average of estimated conditional survival curves from 200 independent replications; and the gray shaded bands are the 90% empirical pointwise confidence bands

**Fig. 3:**
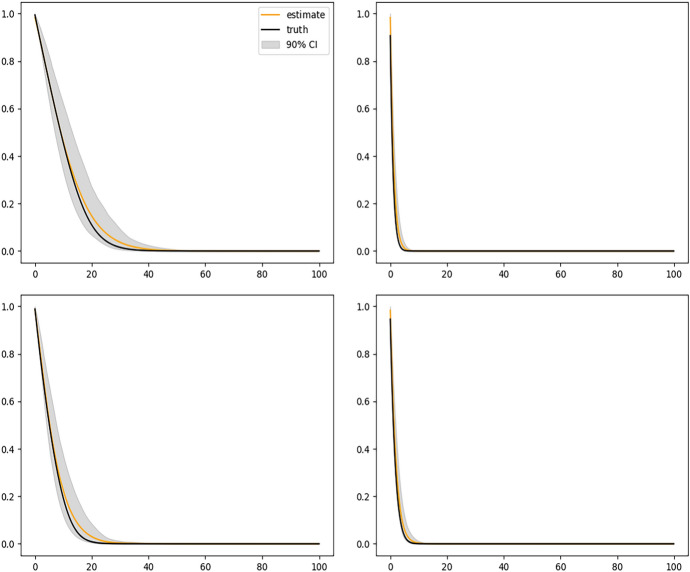
Conditional survival curves for 4 different sets of covariates estimated by the DeepONet with CNN branch net and sample size *n = 2000*. In each plot, black solid curve is the true conditional survival curve; the yellow solid curve is the average of estimated conditional survival curves from 200 independent replications; and the gray shaded bands are the 90% empirical pointwise confidence bands

**Fig. 4:**
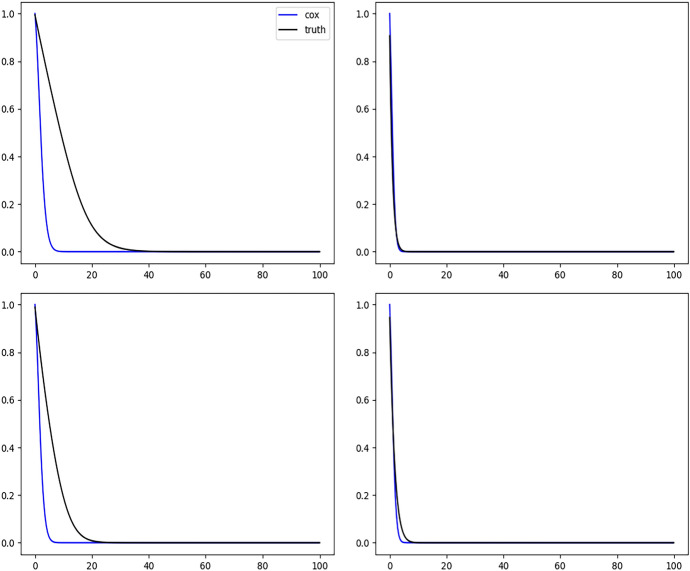
Conditional survival curves for 4 different sets of covariates estimated by the Cox model with sample size *n = 2000*. In each plot, black solid curve is the true conditional survival curve, and the blue solid curve is the average of estimated conditional survival curves from 200 independent replications

Figure 2 shows the results of fitting the DeepONet with FNN branch net and Fig. 3 shows the results with CNN based branch net. We see that the sample average of corresponding estimated curves (solid yellow line) using either method well overlaps with the ground truth (solid black line), but using CNN structure gives slightly narrower confidence bands. Figure 4 shows the biased results obtained from the misspecified Cox model, where only the sample averages are presented. The large bias of the Cox model is expected because the instantaneous covariate effect assumption is violated in this simulation setup.

**Fig. 5:**
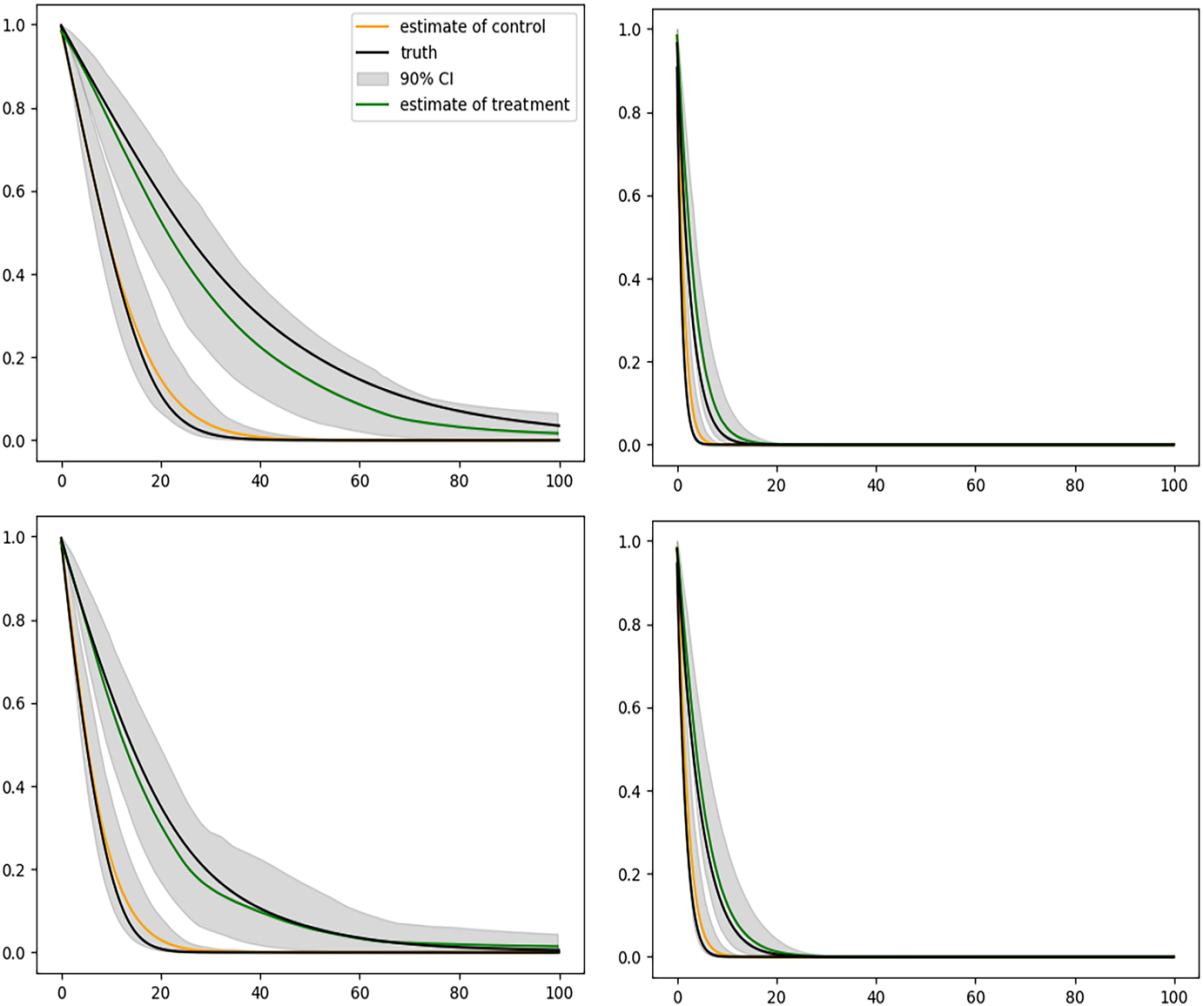
Conditional survival curves corresponding to $z_i = 0$ and $z_i = 1$, respectively, for 4 different sets of covariates $x_i(t)$ and $w_i$, mimicking 4 individuals. In each plot, the solid green curve represents the average of estimated survival curves when $z_i = 1$; the solid yellow curve represents the average of estimated survival curves when $z_i = 0$; and the black solid curves are the corresponding true conditional survival curves

For each of these four sets of covariates, we can obtain two estimated survival curves corresponding to $z_i = 0$ (control group) and $z_i = 1$ (treatment group), respectively, mimicking a setup of estimating the individual level treatment effects nonparametrically given $x_i(t)$ and $w_i$. This also provides a framework of adjusting for potential confounders nonparametrically in causal inference without imposing any model assumptions for the survival data. Figure 5 illustrates such estimates, where the solid green curve represents the sample average of the estimated survival curves when $z_i=1$ and the solid yellow curve represents the sample average of the estimated survival curves when $z_i=0$. The black solid curves are the corresponding true survival curves. We can see that the treatment effect varies among different individuals, reflecting the nature of our simulation setup.

**Fig. 6:**
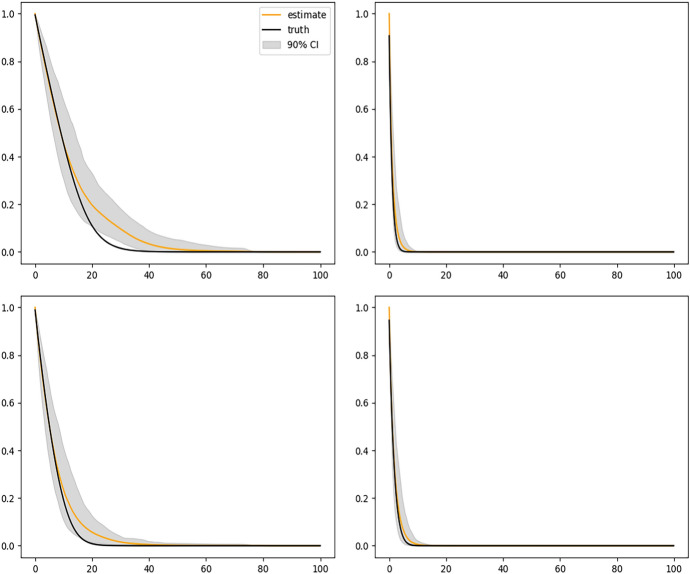
Conditional survival curves for 4 different sets of covariates estimated by the DeepONet with FNN branch net and sample size *n = 500*. Censoring rate is around 40%. In each plot, black solid curve is the true conditional survival curve; the yellow solid curve is the average of estimated conditional survival curves from 200 independent replications; and the gray shaded bands are the 90% empirical pointwise confidence bands

We also perform a simulation study with a smaller sample size and a higher censoring rate. Specifically, we consider a total sample size 1000, of which 80% is used as the training set and the remaining 20% is used as the validation set. Censoring time $C_i$ is generated from an Exponential distribution with mean of 18, truncated at 99. This yields around 40% censoring rate. For the smaller sample size, we choose *m* from $\{50, 100, 200, 300\}$ following the same procedure as before, and obtained *m=200* as the optimal value. The hyperparameters of the neural networks are chosen as follows: the number of nodes in each dense layer (except for the last layer in branch and trunk net) is 128, the learning rate is 0.0001 and batch size is 500. Figure 6 shows the results of fitting the DeepONet with FNN branch net for the same four sets of covariates. The overall patterns are similar to the earlier simulations with a larger sample size and a lower censoring rate, but with slightly more biases. This is expected for a smaller sample size and a higher censoring rate.

## ADNI data analysis

We analyze data from the Alzheimer’s Disease Neuroimaging Initiative (ADNI) (https://adni.loni.usc.edu) to illustrate our method. ADNI data are collected to study the progression of Alzheimer’s disease in the human brain. Specifically, we use ANDIMERGE, which is a merged version of ADNI data, with a data cutoff on January 4, 2025. For our analysis, we select participants who were diagnosed with mild cognitive impairment (MCI) at baseline and who had at least one follow-up visit. The event of interest is defined as the progression to dementia, and the corresponding outcome is progression-free survival. In this context, we consider Mini-Mental State Examination (MMSE) score and hippocampal volume to be external time-varying covariates, and include baseline age as a time-invariant covariate. After excluding individuals with missing baseline MMSE or hippocampal volume, the final dataset consists of observations from 849 individuals. Time-varying covariate values are taken on a six-month interval grid, and missing interim values are imputed using forward-filling. The maximum follow-up duration is 198 months. The failure rate is around 40%.

Given the sparsity of time points in this real-world dataset, we implement the FNN architecture with a single hidden layer as the branch net in our DeepONet framework. The number of nodes in the dense layer is 128, and the learning rate is 0.0001. For comparison, we also fit a Cox proportional hazards model assuming instantaneous time-varying covariate effects. We evaluate the model performance using the integrated Brier score (IBS) (Graf et al. 1999) based on a *K*-fold cross-validation. Specifically, we randomly separate the data set into *K* groups with approximately equal size, which are denoted as $\{\mathcal{D}^{(1)}, \dots , \mathcal{D}^{(K)} \}$. For each fold *k* and *k=1,… , K*, we use $\mathcal{D}^{(k)}$ as the test set and fit the DeepONet using the remaining data $\mathcal{D}^{(-k)}$. The *k*-fold Brier Score is defined as follows:

$$\begin{aligned} \textrm{BS}^{(k)}(t) &= \frac{1}{n_k} \sum _{i \in \mathcal{D}^{(k)}} \Bigg \{\frac{\widehat{S}^{(-k)}(t | x_i)^2I(Y_i \le t, \varDelta _i=1)}{\widehat{G}^{(-k)}(Y_i)} \\ & \quad +\frac{\left[ 1-\widehat{S}^{(-k)}(t | x_i)\right] ^2I(Y_i > t)}{\widehat{G}^{(-k)}(t)} \Bigg \}, \end{aligned}$$

where $\widehat{G}^{(-k)}(t)$ is the Kaplan-Meier estimator of the censoring time survival function using data in $\mathcal{D}^{(-k)}$. Then

$$ \textrm{IBS} = \frac{1}{K}\sum _{k=1}^K \textrm{IBS}^{(k)}, \text{ with } \textrm{IBS}^{(k)} = \frac{1}{Y^{(k)}_{max}-Y^{(k)}_{min}}\int _{Y^{(k)}_{min}}^{Y^{(k)}_{max}} \textrm{BS}^{(k)}(t) dt, $$

where $Y^{(k)}_{min}$ and $Y^{(k)}_{max}$ are the minimum and maximum observed times in the test set $\mathcal{D}^{(k)}$, respectively. We use *K=5* for the ADNI data analysis.

When fitting DeepONet using data in $\mathcal{D}^{(-k)}$, considering the small sample size, we further split 90% of the data in $\mathcal{D}^{(-k)}$ as the training set and the remaining 10% as the validation set. We stop training when validation loss no longer decreases. Our method achieves a lower mean cross-validated IBS of 0.15 compared to 0.28 by the Cox model. The reduction in IBS highlights the effectiveness of our model in capturing complex patterns in the data that the Cox model may not fully account for.

**Fig. 7:**
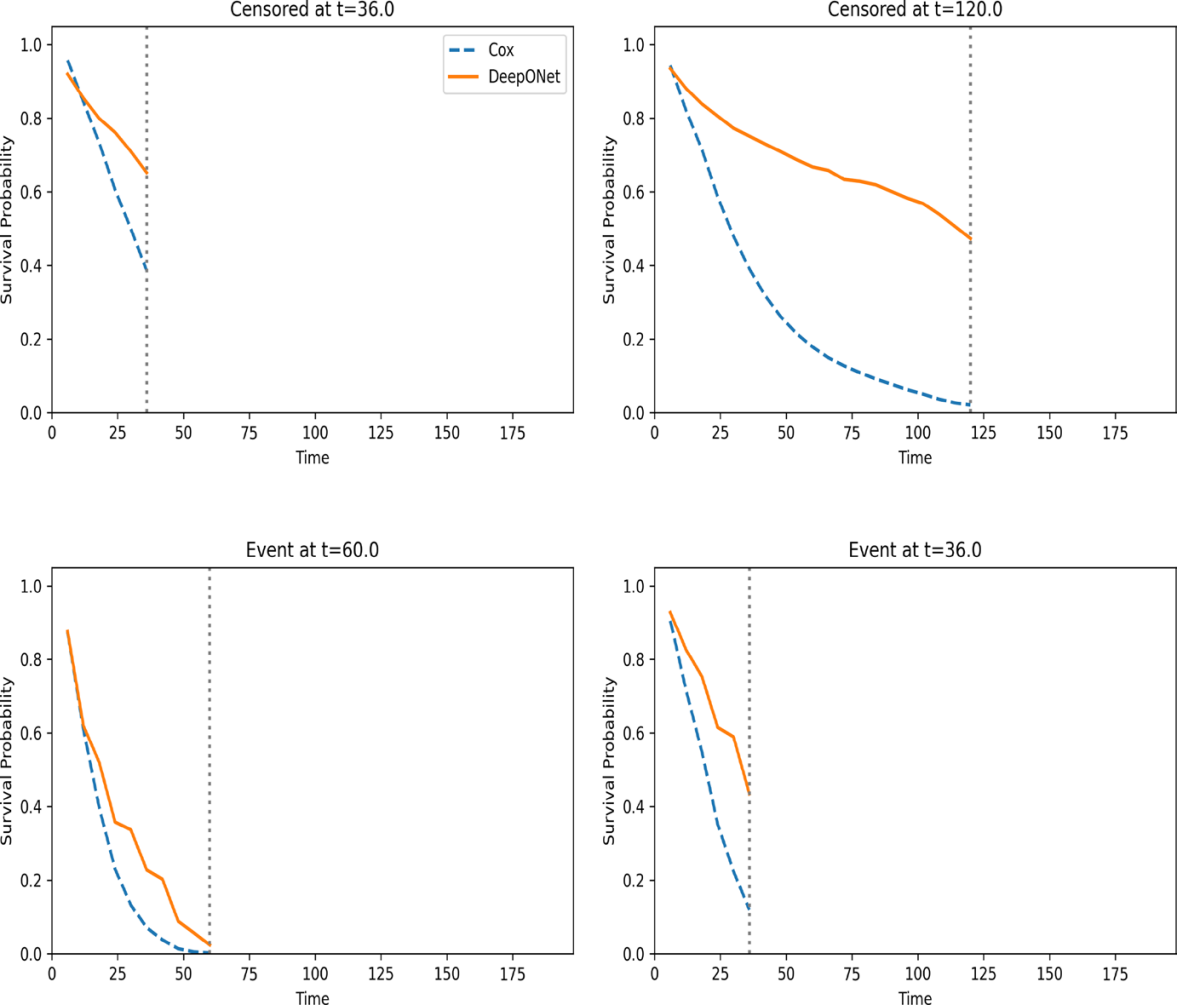
The estimated survival curves using 4 individuals’ covariates. The dotted vertical line represents the observed time for each individual. Time is measured in months

To further illustrate the estimation difference between the DeepONet and the Cox model, we randomly select four individuals and plot the estimated conditional survival functions given their covariate values, where the estimates are obtained using the entire data set. These estimates are plotted in Fig. 7, where we see that these two methods yield very different results.

## Discussion

Our proposed method improves existing machine-learning survival methods in at least two main aspects. First, it is a fully nonparametric approach without imposing any model assumption, whereas methods like DeepSurv (Katzman et al. 2018) retain Cox- or AFT-type structures and estimate only a risk score rather than the full conditional survival function. Second, it treats time-varying covariates as functional inputs, thus allows effects of covariate histories to be arbitrary, whereas all existing methods for time-varying covariates only allow instantaneous covariate effects which can be violated in practice.

Thus, our method flexibly captures complex temporal dynamics and cumulative or delayed effects. To the best of our knowledge, it is the first fully nonparametric method capable of modeling arbitrary time-varying covariate effects in survival prediction with minimal structural assumptions.

A possible limitation of the proposed method is that, when incorporating covariate history into the neural network, we construct an expanded dataset where the covariate history is paired with every time point. This approach, while straightforward, increases the memory requirement due to the large volume of data involved. Therefore, improving the efficiency of data handling and storage is crucial. Future research could focus on developing more computationally efficient representations of covariate histories or incorporating recurrent or attention-based architectures to capture temporal information more efficiently.
