# Correction to: Nonparametric estimation of conditional survival function with time-varying covariates using DeepONet

**DOI:** 10.1007/s10985-026-09724-y

**Published:** 2026-08-14

**Authors:** Bingqing Hu, Bin Nan

**Affiliations:** Department of Statistics, University of California, Irvine, CA 92697 USA

**Correction to: Lifetime Data Analysis (2026) 32:22**

**https://doi.org/10.1007/s10985-026-09700-6**

In this article, there are three errors occurred during production process of the article.

The correction has been provided here and the original article has been corrected.

1. In “Introduction”, the year of Cygu et al. should be 2023 in last sentence of the first paragraph. It should read as given below
  **Corrected sentence**: In particular, Cygu et al. (2023) found that the computational cost is extremely high for RSF when dealing with time-varying covariates.
2. In section “**2.2 Full likelihood and discretized loss”** (on page 6), replace y_i by \infty in two places.
3. The formatting error should be corrected in Table 1.

**Table 1:** The data structure for time-varying covariates

*i*	Time point	Covariates
1	$t_1$	$x_1(t_0),\, 0,\, \dots ,\, 0$
1	$t_2$	$x_1(t_0),\,x_1(t_1),\,0,\, \dots ,\, 0$
⋮	⋮	⋮
1	$t_m$	$x_1(t_0),\, x_1(t_1),\, \dots ,\, x_1(t_{m-1})$
2	$t_1$	$x_2(t_0),\, 0,\, \dots ,\, 0$
2	$t_2$	$x_2(t_0),\, x_2(t_1),\, 0,\, \dots ,\, 0$
⋮	⋮	⋮
2	$t_m$	$x_2(t_0),\, x_2(t_1),\, \dots ,\, x_2(t_{m-1})$
⋮	⋮	⋮

The original article has been corrected.

